# Nicotinamide Augments the Anti-Inflammatory Properties of Resveratrol through PARP1 Activation

**DOI:** 10.1038/s41598-019-46678-8

**Published:** 2019-07-15

**Authors:** Maria Yanez, Megha Jhanji, Kendall Murphy, R. Michael Gower, Mathew Sajish, Ehsan Jabbarzadeh

**Affiliations:** 1 Department of Chemical Engineering, University of South Carolina, Columbia, SC 29208 USA; 2 Biomedical Engineering Program, University of South Carolina, Columbia, SC 29208 USA; 3 Department of Drug Discovery and Biomedical Sciences, College of Pharmacy, University of South Carolina, Columbia, SC 29208 USA

**Keywords:** Biotechnology, Drug discovery

## Abstract

Resveratrol (RSV) and nicotinamide (NAM) have garnered considerable attention due to their anti-inflammatory and anti-aging properties. NAM is a transient inhibitor of class III histone deacetylase SIRTs (silent mating type information regulation 2 homologs) and SIRT1 is an inhibitor of poly-ADP-ribose polymerase-1 (PARP1). The debate on the relationship between RSV and SIRT1 has precluded the use of RSV as a therapeutic drug. Recent work demonstrated that RSV facilitates tyrosyl-tRNA synthetase (TyrRS)-dependent activation of PARP1. Moreover, treatment with NAM is sufficient to facilitate the nuclear localization of TyrRS that activates PARP1. RSV and NAM have emerged as potent *agonists* of PARP1 through inhibition of SIRT1. In this study, we evaluated the effects of RSV and NAM on pro-inflammatory macrophages. Our results demonstrate that treatment with either RSV or NAM attenuates the expression of pro-inflammatory markers. Strikingly, the combination of RSV with NAM, exerts additive effects on PARP1 activation. Consistently, treatment with PARP1 inhibitor antagonized the anti-inflammatory effect of both RSV and NAM. For the first time, we report the ability of NAM to augment PARP1 activation, induced by RSV, and its associated anti-inflammatory effects mediated through the induction of BCL6 with the concomitant down regulation of COX-2.

## Introduction

Inflammation is a process by which our body responds to injuries and attacks pathogens. This process involves various cell types (e.g., neutrophils, T-cells, B-cells, and macrophages), cytokines, and chemokines to restore tissue homeostasis^1,2^. In normal conditions, the process begins with acute inflammation. However, if the immune system fails to resolve the acute inflammatory phase, it can progress to chronic inflammation^3^, which is associated with various degenerative diseases.

Inflammation is commonly treated with non-steroidal anti-inflammatory drugs (NSAIDs), corticosteroids, antibodies, and biological agents. Despite significant progress, negative side effects such as damage to the gastrointestinal track, cardiotoxicity, and hepatotoxicity compromises the effectiveness of these therapeutic strategies^4,5^. The limited clinical benefits of these strategies is rooted in their inability to simultaneously target multiple signaling pathways that regulate inflammation. To this end, there is a critical need for the development of novel multiple-targeting approaches that enable us to modulate the interdependencies of active mediators and ultimately modulate inflammation at a higher clinical efficacy.

Natural products have been a major focus of drug discovery^6–9^. The main inspiration behind their use is the extraordinary ability of natural compounds to target multiple molecular mechanisms in immune, redox, and metabolic regulatory pathways^10,11^. For this reason, resveratrol (RSV), a polyphenolic phytoestrogen found in grapes, nuts, and berries, is increasing in prominence, with reports demonstrating its remarkable potential to induce survival genes that impart cardio-neuro-protective, anti-diabetic, and anti-cancer effects^12–19^. Several published reports have shown that RSV activates SIRT1 and inhibits transcriptional activation of nuclear factor kappa B (NF-κB) resulting in suppression of inflammatory mediators including IL-1β, matrix metallopeptidase 13 (MMP-13), COX-2, IL-6, and TNF-α^13,15,20^. However, two seminal studies published by Sajish and Schimmel, and Park *et al*. have established a SIRT1 independent mechanism of action for RSV through other potential targets, such as mammalian phosphodiesterase-4 (PDE4) and poly-ADP-ribose polymerase-1 (PARP1)^21,22^. Interestingly, PDE4 degrades poly(ADP-ribose) polymer and recycles the ADP-ribose into adenosine monophosphate (AMP) units, resulting in a transient competitive inhibition of cAMP hydrolysis^23–26^. cAMPs regulate the localization, duration, and amplitude of cyclic nucleotide signaling. Consistently, RSV facilitates the activation of PARP1 and accumulation of poly(ADP-ribose) that would lead to ADP-ribose-driven modulation of PDE and subsequent upregulation of cAMP and associated Ca^2+^ signaling^21,22^. These two studies^21,22^ thus suggest that PARP1 and PDE4 act in tandem to exert the metabolic benefits of resveratrol. Because both PARP1 and SIRTuins use NAD^+^, they antagonistically regulate each other, i.e; PARP1 activation inhibits SIRTuins through generation of nicotinamide^22,27,28^ and *vice versa* the metabolic byproducts of nicotinamide (1-Methylnicotinamide)^29,30^ or treatment with nicotinamide riboside (NR) result in the inhibition of PARP1^28,30^ through SIRT1 activation^28,29,31^. Most significantly, inhibition of PARP1 leads to induction of DNA damage and cytotoxicity^32,33^ and mitochondrial dysfunction^34^, which are implicated in the etiology of various metabolic disorders. Although pro-inflammatory interferon gamma (IFN-γ) is known to activate PARP1^35^, prolonged inflammation triggers DNA damage through the inhibition of PARP1^36,37^. Further, PARP1 depletion leads to the sustained induction of a large number of interferon-stimulated genes (ISGs)^38^ and induces senescence^39^. Consistently, emerging works suggest that inhibition of PARP1 induces DNA damage-dependent pro-inflammatory response^36,37,40–42^. These observations suggested that activation of PARP-1 not only enhances DNA repair^43^ but also triggers an anti-inflammatory signaling cascade^36–39^.

Under stress conditions, tyrosyl-tRNA synthetase (TyrRS), which activates L-tyrosine for protein synthesis, translocates to the nucleus and activates PARP1^22^, a major modulator of nicotinamide adenine dinucleotide (NAD^+^) metabolism and signaling. RSV-mediated activation of PARP1 also results in the generation of nicotinamide (NAM)^22^. This leads us to hypothesize that the anti-inflammatory effects of RSV can be mimicked and enhanced upon treatment with NAM. Also known as niacinamide, NAM is a form of vitamin B3 and acts as a classical inhibitor of SIRT1^27,28^. In most mammalian cells, NAM is the main source of NAD^+^, which is necessary for cellular function and energy metabolism^44,45^. NAM has been studied in the context of neuronal diseases, oxidative stress, diabetes, and inflammation^45–50^.

For the first time, we report the ability of NAM to activate PARP1 and its associated anti-inflammatory effects mediated through the induction of BCL6 with the concomitant down regulation of COX-2. Due to their critical role of in immunity and homeostasis, we used macrophages as a testbed for determining the anti-inflammatory signaling of RSV and NAM. The phenotype of macrophages, generally classified as pro-inflammatory (M1 macrophages) and wound healing (M2 macrophages), appears to be a key player in immune response^51–54^. This designation, although conceptually useful, is an oversimplification of the phenotypic diversity of macrophages^55–58^. In reality, macrophages exhibit a complex progression of phenotypes that are affected by local signals as well as the stage of tissue remodeling^59–61^. Significant effort has been dedicated to modulate the phenotypic adaptation of macrophages as a therapeutic target for pathologies^62–65^. To this end, we demonstrate that synergistic activation of PARP1 mediated by RSV and NAM can be exploited as a strategy to tune inflammatory response.

## Results

### Pro-inflammatory macrophage proliferation in the presence of NAM and RSV

**Figure 1:**
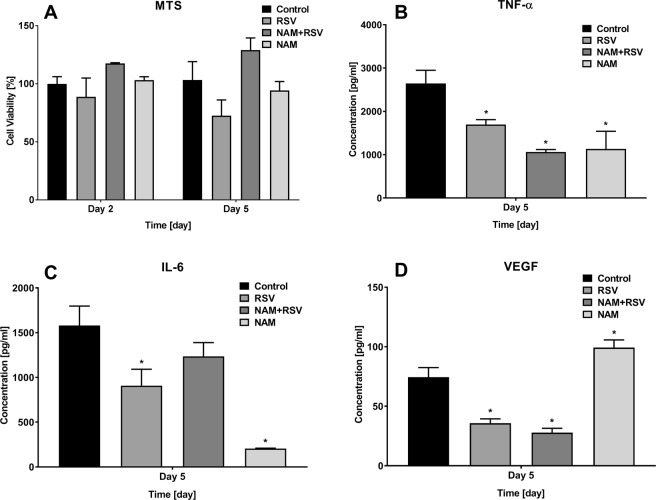
(**A**) Effects of NAM, RSV, and NAM + RSV on cell viability as percentage of untreated control and (**B**–**D**) change in cytokine secretion by ELISA assay. Pro-inflammatory macrophages were incubated with NAM, RSV, or NAM + RSV. (**B**) TNF-α, (**C**) IL-6, and (**D**) VEGF. Data was plotted as mean ± SD (N = 3). *Denotates the (p ≤ 0.05) difference compared to the control group in the same time point. The results demonstrated the potential of NAM and RSV to modulate pro-inflammatory cytokines.

The *in vitro* studies aimed at obtaining a better understanding of the mechanisms that govern the immunomodulatory effects of RSV and NAM on macrophages. We launched our studies by determining the dose response of macrophages to NAM and RSV. Cell viability was determined using a colorimetric assay after the cells were treated with NAM, RSV, their combination (RSV + NAM) at two time points (2 and 5 days). Figure 1A shows the cell viability as compared to non-treated cells (control). We did not observe any adverse effect on macrophage viability when exposed to NAM, RSV, or the combination.

### Pro-inflammatory macrophage cytokine secretion in the presence of NAM and RSV and PARP1 activation

To characterize cytokine secretion of inflammatory macrophages, we assessed the cytokine levels in the supernatants of the treated cells at different time points (2 and 5 days). As expected, LPS-induced inflammatory macrophages produced high levels of TNF-α, IL-6, and VEGF (Fig. 1B–D). The presence of NAM, RSV, and RSV + NAM decreased the LPS dependent induction of pro-inflammatory cytokines TNF-α and IL-6, where the lowest pro-inflammatory cytokine expression level was observed in the NAM treated group (Fig. 1B,C). VEGF expression was also suppressed when pro-inflammatory macrophages were treated with RSV or NAM + RSV, while the NAM-treated macrophages exhibited an increased VEGF expression at day 5 (Fig. 1D).

**Figure 2:**
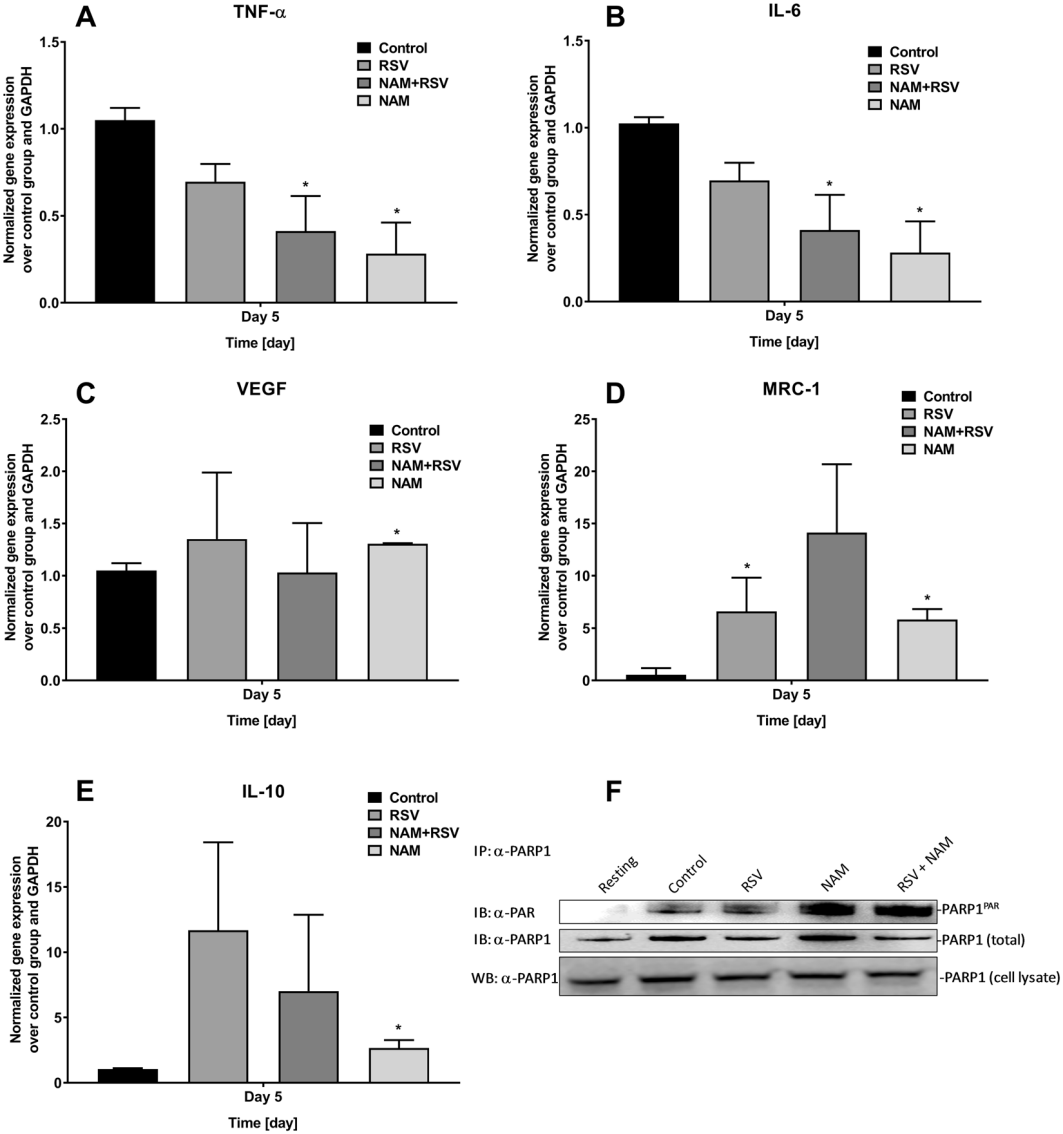
Gene expression changes in pro-inflammatory macrophages over time incubated with NAM, RSV, or NAM + RSV, as analyzed by RT-PCR. Measured genes included (**A**) TNF-α, (**B**) IL-6, (**C**) VEGF, (**D**) MRC-1, and (**E**) IL-10. Gene expression was normalized over the control group and the housekeeping gene GAPDH (2^−ΔΔC^). *Denotes the (p ≤ 0.05) difference compared to the control group in the same time point. (**F**) RSV and NAM synergistically activate PARP1. PARP1 was immunoprecipitated (IP) from activated pro-inflammatory macrophages in the presence of NAM, RSV, or RSV + NAM and immunoblotted (IB) for the presence of poly-ADP-ribose (PAR) using anti-PAR antibody (Millipore). Total PARP1 levels were assessed by anti-PARP1 antibody both by immunoblot and by western blot (WB) in the cell lysate. The cropped blots are displayed in (**F**) and the full-length of blots are presented in Supplementary Fig. 1S.

We assessed cytokine production at the mRNA level using quantitative RT-PCR. The analysis of pro-inflammatory genes (IL-6, TNF-α, and VEGF) and anti-inflammatory genes (MRC-1 and IL-10) demonstrated that NAM, RSV, and RSV + NAM promote anti-inflammatory cytokine expression (Fig. 2A–E). TNF-α was significantly reduced at 5 days in all treated groups (Fig. 2A). Consistently, the inflammatory marker IL-6 was lowered with the addition of 500 μM NAM or 500 μM NAM + 10 μM RSV (Fig. 2B). We observed statistically higher levels of VEGF gene expression for the group containing NAM at day 5 (Fig. 2C). MRC-1 levels increased in all treated groups (Fig. 2D). In addition, IL-10 levels were significantly higher for the inflammatory macrophages cultured with RSV + NAM, as compared to the control group with no compounds (Fig. 2E). And at day 5, all treated groups demonstrated higher IL-10 as compared to the control non-treated inflammatory macrophages. The highest level of IL-10 expression was observed in macrophages exposed to RSV (Fig. 2E). Further, we measured the activation of PARP1 in pro-inflammatory macrophages treated with RSV and NAM by monitoring the induction of poly-ADP-ribose (PAR) levels. Figure 2F demonstrates that NAM activates PARP1 more robustly than RSV. The results all together suggested that NAM and RSV facilitates the activation of PARP1 and stimulates a PARP1-dependent anti-inflammatory signaling cascade^22,36,38^.

### PARP1 inhibition in pro-inflammatory macrophages in response to NAM and RSV

**Figure 3:**
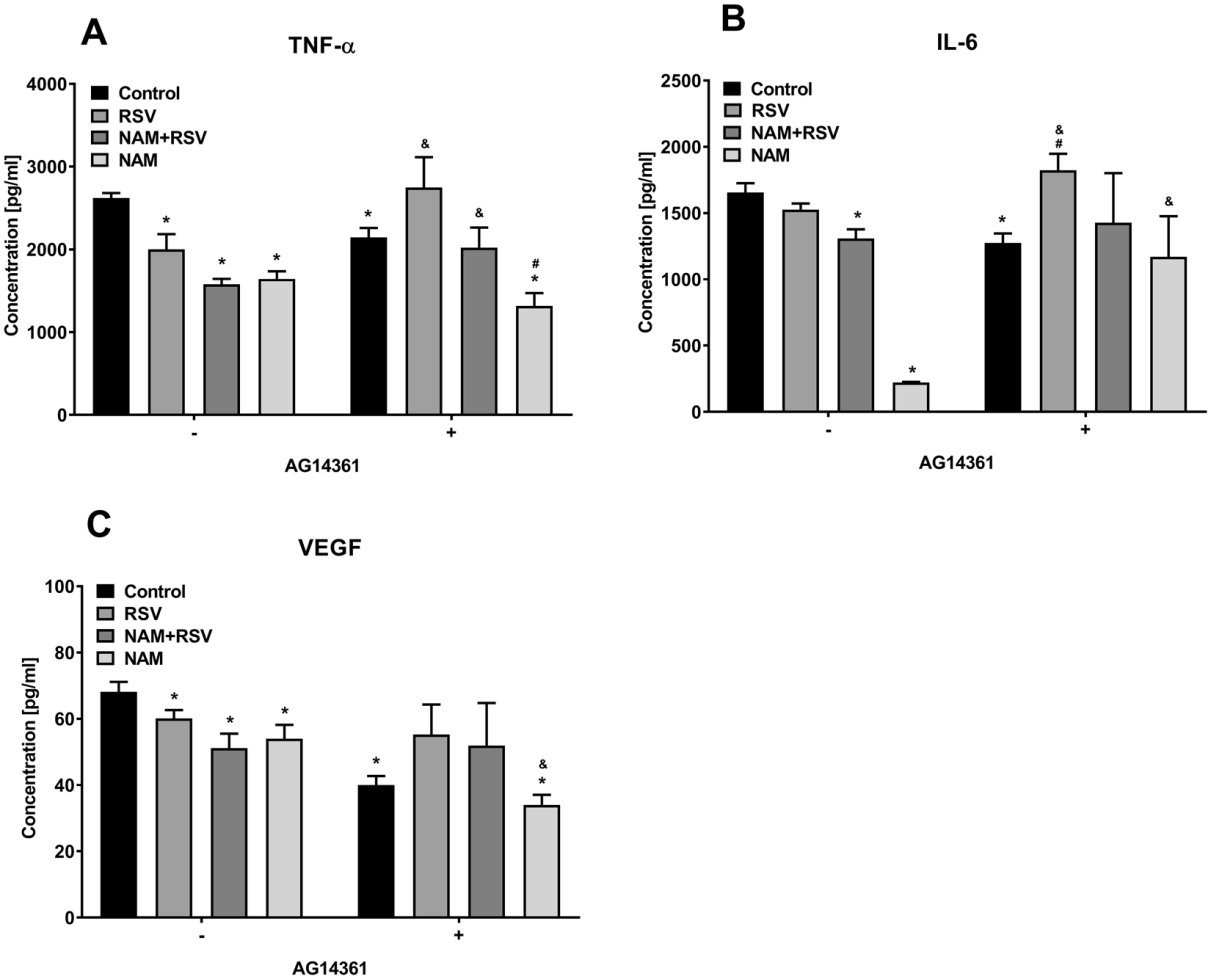
Change in cytokine secretion over time. The PARP1 pathway was blocked by the addition of AG14361 inhibitor to the pro-inflammatory macrophages. Then the cells were treated with NAM, RSV, or NAM + RSV, and the supernatant was collected 48 hours later for analysis by ELISA. (**A**) TNF-α, (**B**) IL-6, and (**C**) VEGF. ^*^Denotes the (p ≤ 0.05) difference compared to the control group in the same time point. ^#^Denotes the (p ≤ 0.05) difference for all AG14361 treated groups compared to the control + AG14361 group. & denotes the (p ≤ 0.05) difference between the same treatment in the presence or absence of AG14361 at the same time point.

**Figure 4:**
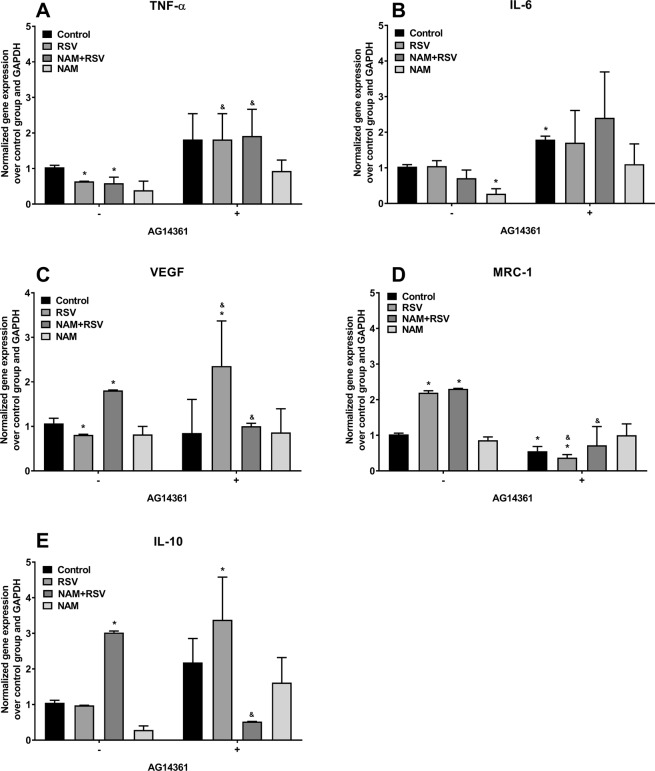
Gene expression of pro-inflammatory macrophages incubated with NAM, RSV, or NAM + RSV in the presence (+) or absence (−) of AG14361 (PARP1 inhibitor). RNA was extracted after 2 days and analyzed by RT-PCR. (**A**) TNF-α, (**B**) IL-6, (**C**) VEGF, (**D**) MRC-1, and (**E**) IL-10. Gene expression was normalized over the control group and the housekeeping gene GAPDH (2^−ΔΔC^). ^*^Denotes the (p ≤ 0.05) difference compared to the control group in the same time point. ^#^Denotes the (p ≤ 0.05) difference for all AG14361 treated groups compared to the control + AG14361 group. & denotes the (p ≤ 0.05) difference between the same treatment in the presence or absence of AG14361 at the same time point.

To further establish the role of PARP1 in RSV- and NAM-mediated anti-inflammatory effects, we added a chemical inhibitor of PARP1 (AG14361) to the culture media of the LPS-induced inflammatory macrophages. Supernatant and RNA was collected after 2 days of treatment. Cytokine expression in the supernatant was determined by ELISA. Levels of pro-inflammatory cytokines in the cell culture media were lower for all groups except those treated with PARP1 inhibitor, as compared to the non-knockdown group. We observed that AG14361 reversed the anti-inflammatory effects of NAM, RSV, or RSV + NAM as demonstrated by higher expression levels of TNF-α, IL-6 (Fig. 3A–C). We performed RT-PCR to confirm the cytokine expression via mRNA levels when the LPS-induced inflammatory macrophages were exposed to AG14361 PARP1 inhibitor. As demonstrated in Fig. 4A, TNF-α expression was consistently higher in all groups when compared to the group treated with pro-inflammatory LPS media free of AG14361. The same effect was observed for IL-6 (Fig. 4B). VEGF expression was the same for all groups (Fig. 4C). The anti-inflammatory cytokine expression of MRC-1 was not as enhanced for the NAM, RSV, or RSV + NAM treated samples, which were exposed to AG14361 (Fig. 4D) when compared to those same groups in the absence of AG14361, while the IL-10 cytokine expression was enhanced for almost all the groups treated with AG14361 with the exception of the NAM + RSV group (Fig. 4E). These findings revealed that the pro-inflammatory and anti-inflammatory effects of these mediators (IL-6, TNF-α, VEGF, MRC-1, and IL-10) are regulated by PARP1.

**Figure 5:**
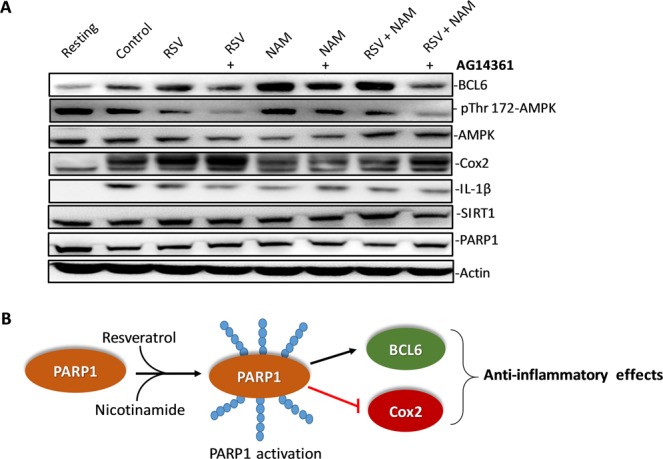
PARP-1 inhibitor antagonizes the anti-inflammatory effects of RSV by down-regulating the expression of BCL6 and upregulating COX-2. (**A**) AG14361 PARP-1 inhibitor was added to the pro-inflammatory polarization media in the presence or absence of NAM, RSV, or RSV + NAM. “+” indicates that AG14361 was added. Samples were processed and western blotted for the expression level of BCL6 and COX-2. The cropped blots were displayed in (**A**) and the full-length of blots were presented in Supplementary Fig. 2S. (**B**) Proposed mechanism of PARP-1-mediated anti-inflammatory effects of RSV and NAM.

B-cell lymphoma-6 protein (BCL6) is a corepressor for inflammatory mediators that recruits monocytes to vascular endothelial cells upon inflammation. Because PARP1 mediated expression of BCL6 is known to play an anti-inflammatory role^66^, we checked the induction of BCL6 after RSV and NAM treatment and observed a strong induction of BCL6 (Fig. 5). More interestingly, we observed that similar to RSV/PARP1-dependent AMPK activation^22,67^, NAM also triggered a profound activation of 5′ AMP-activated protein kinase (AMPK) which is also a well-known negative regulator of NF-kB^68^. Further, consistent with its known anti-inflammatory effect^48^ and PARP1-mediated inhibition of COX-2^69^, treatment with NAM also resulted in the profound down regulation of COX-2, a well-known mediator of inflammation. Finally, treatment with AG14361- a potent PARP1 inhibitor, abrogated NAM + RSV mediated AMPK activation, Bcl-6 induction and COX-2 down regulation (Fig. 5). Therefore, this work demonstrated that PARP-1 is critical for the RSV and NAM-mediated anti-inflammatory effects.

## Discussion

The imbalance of cytokines, chemokines, and some other mediators can affect cell differentiation and disturb cellular homeostasis. All these changes influence cell metabolism and change tissue functions that may favor disease progression. Various treatments have been applied to treat chronic inflammation, including antibodies, biological agents, and NSAIDs, which target different pro-inflammatory mediators, such as TNF-α, IL-6, IL-1β, and COX, to name a few^70^. However, these treatments can results in different side effects, like gastritis, ulcers, colitis, bleeding, hepato-renal dysfunction, organ failure, and skin dysfunction^71,72^. As a result, there has been increasing interest in treatments derived from natural products with minimal side effects.

RSV is among the natural compounds that have demonstrated unique potential suppress the inflammatory process by downregulating pro-inflammatory cytokine secretion and improve health^17,73–75^. Previous studies have shown that RSV acts through NAD^+^ dependent proteins, and play an important role in PARP1 activation^22^. NAM is a precursor of NAD^+^ that prevents the degradation of PARP1 and protects cells by maintaining DNA integrity and preventing acute cellular degradation^76^. Thus, we hypothesized that the anti-inflammatory effects of RSV can be enhanced upon treatment with NAM.

We began this work by designing a series of *in vitro* experiments using pro-inflammatory macrophages to confirm the anti-inflammatory effect and the molecular mechanism of action of RSV and NAM. The results obtained in this study provide information on baseline cytokine profiles of LPS-induce pro-inflammatory macrophages, and the anti-inflammatory effects of NAM and RSV in this system. Consistent with a previous published report^46^, we observed that both NAM and RSV could suppress the expression of pro-inflammatory cytokines, including TNF-α and IL-6 and could up-regulate the expression levels of the anti-inflammatory mediators (IL-10 and MRC-1). Strikingly, the effects of NAM were more profound as compared to RSV alone; while the co-treatment with RSV further modulated the effects of NAM. The results suggest that a combination of RSV and NAM helps trigger the conversion of NAD^+^ through the salvage pathway and result in a favorable immune response.

We hypothesized that the anti-inflammatory effect of RSV and NAM was related to AMPK and PARP1 activation. We knew that SIRT1 did not play any role in this anti-inflammatory effect because NAM has been used as a potent SIRT1 inhibitor^27,50^, but we were not sure about the AMPK and PARP1. To test this hypothesis, we used a PARP1 inhibitor (AG14361) with established potency in previous studies^22,77^ in the presence or absence of RSV and/or NAM to assess. Our results suggest a novel mechanism of action mediated through PARP1 -dependent induction of BCL6^66^ with concomitant inhibition of COX-2^69,78^ that is responsible for the observed anti-inflammatory effects of RSV^78^ and NAM. Our work is also consistent with the published study by Pantano *et al*. reporting that in LPS stimulated human macrophages the basal levels of BCL6 disappear right after LPS treatment and was fully restored between 1 and 2 hours post-treatment^79^. This work thus provides the molecular basis for the newly emerging PARP1-mediated anti-inflammatory signaling cascade^36–39^.

## Conclusions

A single dose of RSV, NAM, or RSV + NAM attenuated inflammation by suppressing pro-inflammatory cytokines released in LPS-induced mouse and LPS-treated macrophages through PARP1 activation. Therefore, like RSV, NAM is also a potent activator of PARP1. Furthermore, this work demonstrates that beyond facilitating DNA repair, PARP1 activation also triggers anti-inflammatory effects. The present study thus suggests that the synergistic effect of RSV and NAM on PARP1 activation would provide a potential therapeutic strategy to treat inflammatory diseases.

## Materials and Methods

### Monocyte culture, differentiation to M0, and polarization to pro-inflammatory macrophages

Human monocytic cells (THP-1 cells) were obtained from the American Type Culture Collection (ATCC) and cultured in RPMI-1640 media supplemented with 10% fetal bovine serum (FBS), 1% penicillin-streptomycin (PS), and 0.05 mM 2-mercaptoethanol. THP-1 cells were differentiated into M0 macrophages by culturing the cells with 100 ng/ml of 12-myristate 13-acetate (PMA) (Sigma) for 24 hours^17,80,81^. After differentiation, the cells were washed three times with serum free culture media to remove non-differentiated cells. To activate M0 macrophages, the cells were exposed to polarization media, which consisted of culture media supplemented with 100 ng/ml of lipopolysaccharide (LPS) (Sigma) from *Escherichia Coli* serotype 0111:B4 and 20 ng/ml of interferon (IFN)-γ (Peprotech). To assess the effects of NAM, RSV, and the combination of RSV and NAM (RSV + NAM), pro-inflammatory macrophages were exposed to RSV (10 µM), NAM (500 µM) or RSV (10 µM) plus NAM (500 µM) for durations of 16, 24, 48, and 120 hours.

### Analysis of pro-inflammatory macrophage proliferation in the presence of NAM and RSV

Cell proliferation was assessed using the CellTitter 96 Aqueous Non-Radioactive Cell Proliferation assay (MTS assay, Promega)^82^, which is based on the redox conversion of a tetrazolium salt into formazan product. Briefly, THP-1 cells were seeded, differentiated to M0 macrophages, and polarized to pro-inflammatory macrophages in 96-well plates (5 × 10^4^ cells/well). Five groups were considered: (i) activated macrophages in polarization media (control), and pro-inflammatory macrophages in (ii) polarization media, (iii) polarization media including 500 µM of NAM, (iv) polarization media including 10 µM of RSV, or (v) polarization media including 500 µM NAM plus 10 µM of RSV. We monitored the cells at time points of 2 and 5 days. At the end of each exposure time point, MTS solution was added to the cells following the kit’s instruction and the samples were incubated for 2 hours at 37 °C. The absorbance was determined using a Spectramax 190 microplate spectrophotometer at 490 nm. The results were expressed as percent cell viability compared to the control.

### Analysis of pro-inflammatory macrophage cytokine secretion in the presence of NAM and RSV

THP-1 cells were seeded, differentiated from monocytes to M0 macrophages, and polarized to pro-inflammatory macrophages in 12-well plates (5 × 10^5^ cells/well). Inflammatory macrophages were exposed to polarization media containing either (i) 500 µM of NAM, (ii) 10 µM of RSV, or (iii) 500 µM NAM plus 10 µM of RSV for time points of 2 and 5 days. Next, the supernatant was collected, centrifuged, and stored at −20 °C. Cytokine secretion (TNF-α, IL-6, and VEGF) in the culture media was assessed using an enzyme-linked immunosorbent assay (ELISA) kit following the manufacturer’s protocols (Peprotech). To quantify, calibration curves for TNF-α, IL-6, and VEGF standards were generated. Colorimetric changes were measured using a SpectraMax 190 microplate spectrophotometer at 450 nm with a wavelength correction set at 620 nm.

### Analysis of pro-inflammatory macrophage phenotypic response to NAM and RSV

We assessed the expression of pro- and anti-inflammatory genes expressed by macrophages, including TNF-α, IL-6, VEGF, MRC-1, and IL-10, using quantitative reverse transcription polymerase chain reaction (RT-PCR). Inflammatory macrophages were exposed to polarization media containing either (i) 500 µM of NAM, (ii) 10 µM of RSV, or (iii) 500 µM NAM plus 10 µM of RSV for time durations of 2 and 5 days. Total RNA was isolated using the Gene Jet RNA Purification kit (Thermo Scientific). The quantification total of RNA was performed using a Nanodrop 2000c spectrometer and considered pure if the ratio of the absorbance at 260 nm/280 nm was ≥2. The RNA was prepared as a template for cDNA synthesis using the iScript cDNA synthesis kit (Bio-Rad). Quantitative RT-PCR was performed using 10.4 ng of cDNA per reaction and SYBER® Green PCR Supermix (Bio-Rad). Gene expression was normalized to the housekeeping gene GAPDH and the control group (pro-inflammatory macrophages in polarization media). Gene expression values were calculated using the mean C_t_ values of the samples. All primers (Table 1) were synthetized by Integrated DNA Technologies.

**Table 1:** Primers used for quantitative real-time polymerase chain reaction.

Gene	5′-3′ primer sequences: (F: forward R: reverse)
VEGFA	F: AGC CTT GCC TTG CTG CTC TA
	R: GTG CTG GCC TTG GTG AGG
MRC-1	F: CAG CGC TTG TGA TCT TCA TT
	R: TAC CCC TGC TCC TGG TTT TT
IL-10	F: GTG ATG CCC CAA GCT GAG A
	R: CAC GGC CTT GCT CTT GTT TT
TNFα	F: CTG CTG CAC TTT GGA GTG AT
	R: AGA TGA TCT GAC TGC CTG GG
IL-6	F: AGC CAC TCA CCT CTT CAG AAC
	R: GCC TCT TTG CTG CTT TCA CAC
GAPDH	F: GTG GAC CTG ACC TGC CGT CT
	F: GGA GGA GTG GGT GTC GCT GT

### Analysis of PARP1 activation in pro-inflammatory macrophages in response to NAM and RSV

The activation of PARP1 in pro-inflammatory macrophages treated with RSV, NAM, or both was monitored by measuring downstream signaling markers associated with the NAD+ (substrate for sirtuins and PARP signaling) metabolic flux. Targets were selected if they were activated and/or inhibited under conditions of RSV and NAM treatment stress or NAD^+^ metabolic byproduct-treatment. For example, the induction of NAMPT (a major regulator of NAD^+^ levels in cells) is a downstream signaling event of PARP1 activation^22^. Similarly, PARP1 activation induces phosphorylation of H2B (substrate for AMPK, which upregulates NAMPT expression)^83^.

Inflammatory macrophages were exposed to polarization media containing either (i) 500 µM of NAM, (ii) 10 µM of RSV, or (iii) 500 µM NAM plus 10 µM of RSV for 24 hours. Cells were rinsed with ice-cold PBS and ice-cold lysis buffer. Next, cells were scrapped off from the tissue culture well-plates, collected, and processed in order to generate whole cell lysate. The whole cell lysate was processed further for western blot analysis using an iBLOT system (Invitrogen). Blots were developed using antibodies and detected using a BioRad Chemi-Doc imaging system. For immunoprecipitation, the supernatants were pre-cleared by incubation with protein G beads. The pre-cleared cell lysates were incubated at 4 °C for 1 hour with either α-PARP1, α-TyrRS, or non-immune immunoglobulin-G (IgG) at a concentration of 5 mg/ml followed by incubation in 30 ml of Protein G beads (pretreated with 10 mg/ml BSA) at 4 °C for 1 hour. Immunoprecipitates were washed three times, subjected to SDS-PAGE, and immunoblotted with antibodies.

To further elucidate the role of PARP1 in anti-inflammatory effects of RSV and NAM on pro-inflammatory macrophages, inflammatory macrophages (5 × 10^5^ cells/ml) were treated with 10 µM of AG14361 (a potent inhibitor of PARP1). The experimental design included four conditions: (i) polarization media (control), (ii) polarization media containing 500 µM of NAM, (iii) polarization media containing 10 µM of RSV, and (iv) polarization media containing 500 µM NAM plus 10 µM of RSV. The negative control was carried out using inflammatory macrophages cultured in polarization media without the PARP1 inhibitor. Cytokine (TNF-α, IL-6, and VEGF) as well as gene expression (TNF-α, IL-6, VEGF, MRC-1, and IL-10) were evaluated using an ELISA assay and quantitative RT-PCR, respectively. Immunoblotting was used to evaluate the BCL6 and AMPK activation 24 hours following the treatment.

### Statistical analysis

All the experiments were performed in duplicates. A minimum of three samples (N = 3) were analyzed per condition unless otherwise stated. Data are presented as mean ± standard deviation (SD). Statistical analysis was done by using GraphPad Prism 7. Multiple Student’s t test was performed to evaluate differences between treated groups and control groups. Statistical significance was defined as p ≤ 0.05.

## Supplementary information

Supplementary data

## Data Availability

All relevant data are within the paper.
