# Supplementary material for: Nicotinamide Augments the Anti-Inflammatory Properties of Resveratrol through PARP1 Activation
Source: Sci Rep. 2019 Jul 15;9:10219. doi: 10.1038/s41598-019-46678-8 (PMC6629694; doi:10.1038/s41598-019-46678-8)
Supplement: Supplementary file 1 — Supplementary data [file 41598_2019_46678_MOESM1_ESM.docx]

**Supplementary information**

**Nicotinamide Augments the Anti-Inflammatory Properties of Resveratrol through PARP1 Activation**

Maria Yanez^1^, Megha Jhanji^3^, Kendall Murphy^1^, R. Michael Gower^1,2^, Mathew Sajish^3^ and Ehsan Jabbarzadeh^*1,2^

^1^Department of Chemical Engineering, University of South Carolina, Columbia, SC, 29208, USA

^2^Biomedical Engineering Program, University of South Carolina, Columbia, SC, 29208, USA

^3^Department of Drug Discovery and Biomedical Sciences, College of Pharmacy, University of South Carolina, Columbia, SC 29208, USA

**
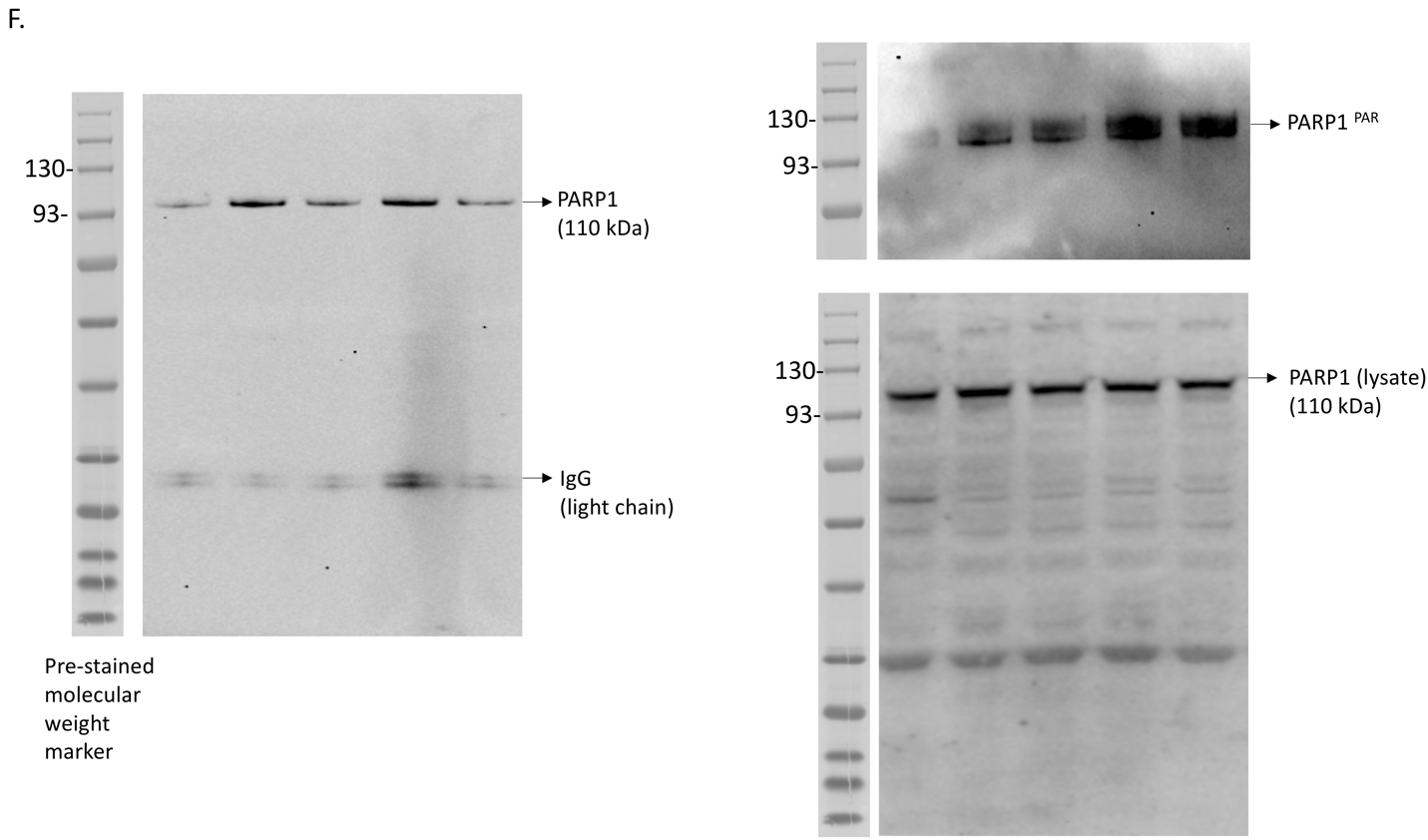
**

**Figure 1S.** RSV and NAM synergistically activate PARP1. PARP1 was immunoprecipitated (IP) from activated pro-inflammatory macrophages in the presence of NAM, RSV, or RSV+NAM and immunoblotted (IB) for the presence of poly-ADP-ribose (PAR) using anti-PAR antibody (Millipore). Total PARP1 levels were assessed by anti-PARP1 antibody both by immunoblot and by western blot (WB) in the cell lysate. The cropped blots are displayed in (F) . Only one gel was used for the imaging, and the antibodies used for this experiment are presented in Table 1S.

**
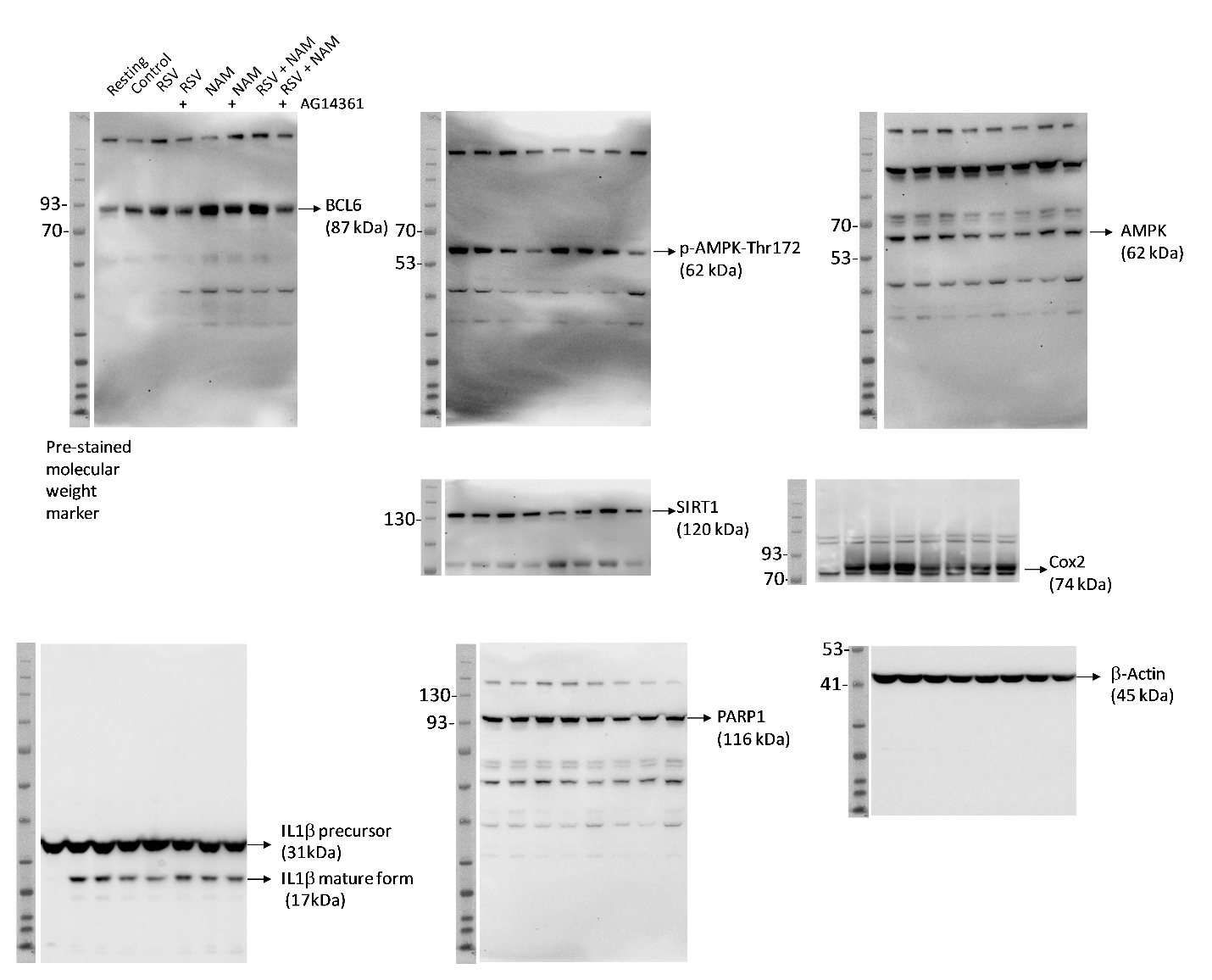
**

**Figure 2S.** PARP1 inhibitor antagonizes the anti-inflammatory effects of RSV by down-regulating the expression of BCL6 and upregulating COX-2. AG14361 PARP1 inhibitor was added to the pro-inflammatory polarization media in the presence or absence of NAM, RSV, or RSV+NAM. “+” indicates that AG14361 was added. Samples were processed and the expression of BCL6 and COX-2 were determined by western blot. Images were taken from the same samples run in duplicates in two gels. The antibodies used for this experiment are presented in Table 1S.

**Table 1S.** Antibodies used for the blots

| **Antibodies** | **Catalog Number** | **Supplier** |
| --- | --- | --- |
| Cox2 (D5H5) XP® Rabbit mAb | 12282S | Cell signaling technology |
| IL-1β (3A6) Mouse mAb | 12242S | Cell signaling technology |
| AMPKα | 2532 | Cell signaling technology |
| Phospho-AMPKα | 2535 | Cell signaling technology |
| PARP1 antibody | 46D11 | Cell signaling technology |
| PADPR mouse Mab | ab14459 | AbCam |
| SIRT1 | 9475 | Cell signaling technology |
| β-Actin (8H10D10) Mouse mAb | 3700 | Cell signaling technology |
| BCL6 (D4I2V) Xp rabbit mAb | 14895 | Cell signaling technology |
